# Structure and Immunogenicity of the *Bordetella pertussis* LOS-Derived Oligosaccharides in the Endosomal-Like Pre-Processing Mice Model

**DOI:** 10.3390/vaccines9060645

**Published:** 2021-06-13

**Authors:** Sabina Koj, Karolina Ucieklak, Czeslaw Lugowski, Tomasz Niedziela

**Affiliations:** Hirszfeld Institute of Immunology and Experimental Therapy, 53-114 Wroclaw, Poland; sabina.koj@hirszfeld.pl (S.K.); karolina.ucieklak@hirszfeld.pl (K.U.); czeslaw.lugowski@hirszfeld.pl (C.L.)

**Keywords:** *Bordetella pertussis*, lipooligosaccharide, core oligosaccharide, neoglycoconjugates, endosomal glycan processing, reactive nitrogen species, vaccine design

## Abstract

Glycoproteins are processed endosomally prior to presentation to T cells and subsequent induction of specific antibodies. The sugar part of glycoconjugate may be degraded while the type of the process depends on the features of the particular structure. The generated carbohydrate epitopes may differ from native structures and influence immunogenicity of the antigens. We have devised a model of endosomal-like pre-processing of *Bordetella pertussis* 186 oligosaccharides (OSs) to verify how it affects the immunogenicity of their conjugates. The glycoconjugates of structurally defined forms of the dodecasaccharide OS were synthesized and their immunogenicity was assessed using immunochemical methods. The structural features of the oligosaccharides and their sensitivity to deamination were analyzed by NMR spectroscopy. The distal trisaccharide-comprising pentasaccharide conjugated to a protein was the most effective in inducing immune response against the *B. pertussis* 186 LOS and the immune response to the complete OS conjugates was significantly lower. This could be explained by the loss of the distal trisaccharide during the in-cell deamination process suggesting that the native structure is not optimal for a vaccine antigen. Consequently, our research has shown that designing of new glycoconjugate vaccines requires the antigen structures to be verified in context of possible endosomal reactions beforehand.

## 1. Introduction

Pertussis is a highly contagious disease involving the respiratory tract and is especially dangerous for infants and young children. Widespread immunization has significantly reduced the overall number of cases in many countries [1]. However, the number of pertussis cases has been on the rise recently, and especially among adolescents and adults [2,3,4,5,6]. Adverse effects of immunizations with the whole-cell pertussis vaccines led to the development of acellular pertussis vaccines composed of different surface proteins, such as filamentous hemagglutinin, pertactin, fimbriae and inactivated pertussis toxin [7,8,9,10,11]. Today, different formulations of acellular vaccines are available, but their efficacy at most reaches 80–85%. It has been reported that the prolonged use of acellular vaccines effects the pertussis protein antigens in the wild, contributes to the observed antigenic drift and leads to a vaccine-pathogen mismatch. As a result a number of pertussis incidents has been on the rise [12,13].

Among the virulence factors of *B. pertussis*, endotoxin is not considered as a potential candidate for a vaccine component due to its toxicity. Lipopolysaccharide (LPS) is the most abundant cell surface molecule of *B. pertussis* and plays a major role in the host-pathogen interactions [14]. It is also responsible for endotoxic activities similar to those of enteric bacteria [15,16]. *B. pertussis* produces only two types of LPS [17], comprising either a nonasaccharide or alternatively a dodecasaccharide linked to a lipid A moiety. In the LPS of *B. pertussis* the O-specific polysaccharide chain is replaced by a single distal trisaccharide and therefore structurally it constitutes a lipooligosaccharide (LOS).

The biological activities of *B. pertussis* LOS are similar to those of endotoxins among other Gram-negative bacteria. The lipid A of LOS contributes to activation of macrophages and stimulation of TNF-α production. LOS is also an important etiological factor in whooping cough. Working synergistically with tracheal cytotoxin and pertussis toxin, LOS takes part in induction of nitric oxide synthase in nonciliated epithelial cells, followed by NO release and subsequent damage to the ciliated cells of the human airways mucosa [18,19]. Due to these toxic effects intact LOS cannot be included as a component in pertussis vaccines.

On the other hand, LOS of *B. pertussis* is an antigen in natural infection [20]. Antibodies specific for the dodecasaccharide-containing LOS of *B. pertussis* have complement-dependent bactericidal activity and substantially reduce colonization of the respiratory tract of mice following aerosol infection [21]. Moreover, the oligosaccharide segment of *B. pertussis* LOS contains the epitopes for the bactericidal antibodies [20,22]. However, the oligosaccharides alone are not immunogenic and thus they are not suitable as vaccine components. To circumvent this lack of immunogenicity of the oligosaccharides the neoglycoconjugates can be synthesized [23,24]. When the oligosaccharide is covalently linked to a protein carrier it becomes immunogenic. In case of *B. pertussis,* it is of utmost importance that the oligosaccharide segment of LOS is evolutionary stable and remains virtually unchanged among clinical isolates over the years [22,25].

Acellular pertussis vaccines currently available act solely by toxin neutralization that could contribute to their limited efficacy. Deprived of oligosaccharide epitopes of *B. pertussis* LOS, the vaccines do not induce production of opsonophagocytic antibodies that are necessary to destroy and eliminate the bacteria. Preliminary investigation of the glycoconjugates of oligosaccharide fragments derived from *B. pertussis* LOS with suitable protein carriers (pertussis toxin, tetanus toxoid) demonstrated that immunization of animals (rabbits) induced production of antibodies that were specific for the carbohydrate segment and showed distinct bactericidal activity in in vitro tests [26,27,28]. These observations imply that non-toxic oligosaccharide segments of *B. pertussis* LOS constitute a potential additional component of acellular vaccines.

The complete oligosaccharide segment of *B. pertussis* LOS includes the core oligosaccharide and a distal trisaccharide [26,29]. However, when conjugated with a protein the oligosaccharides derived from such dodecasaccharide may expose different immunodominant regions. The structures of final epitopes could be governed by carbohydrate-specific cellular processes. Therefore, it would be advantageous for the design of carbohydrates as potential vaccine components to get an insight into the relation of the structure of oligosaccharide used for conjugation and the observed immunogenicity.

As shown by Cobb et al., Duan et al. and Sun et al. glycoconjugates undergo processing in endosomes prior to being presented to T-cells [30,31,32,33,34]. Both protein and carbohydrate parts are processed, however the mechanisms differ. While proteins are typically subjected to enzymatic proteolysis, glycan segments require an action of reactive-nitrogen-species (RNSs) and reactive-oxygen-species (ROSs). It was demonstrated that a contact of carbohydrate molecules with the antigen-presenting-cells (APC) leads to an increased expression of inducible NO synthase (iNOS). Subsequently a reactive-nitrogen is formed, resulting in the modification of the saccharide residues that are susceptible to deamination. The susceptible polysaccharide is degraded by RNSs into fragments with lower molecular mass, still covalently coupled to peptide fragment of protein carrier and such moieties are subsequently bound to MHC II and presented to T lymphocytes. The activated T cells interact with B lymphocytes, which in turn are stimulated to produce glycan-fragment-specific antibodies and provide the memory IgG response as well. Therefore, it was concluded that the polysaccharide processing is a crucial step for generating the sugar epitopes that effectively leads to specific anti-oligosaccharide antibodies production.

The deamination is considered the dominant mode of carbohydrate endosomal processing [30,31,32,33]. Therefore, we have hypothesized that *B. pertussis* oligosaccharide (OS) would be suitable for analysis of the effects of the endosomal-like oligosaccharide processing monitored by the assessment of the induced immunogenicity.

The complete *B. pertussis* core OS contains glucosamine residues with non-acetylated amine groups which are susceptible to deamination. In our project, the OS was degraded in a controlled way to obtain the “OS-deaminated” which is a distal trisaccharide-comprising pentasaccharide in processes that mimic the effects of RNSs obtained from nitric oxide. Subsequently, neoglycoconjugates of the LOS-derived oligosaccharides were prepared and their immunogenicity was assessed. Thus, we have devised a model of endosomal-like pre-processing of *B. pertussis* 186 OS glycoconjugates and assumed that deamination is its dominant mode. Herein we report on the structural details in relation to the observed immunogenicity for the most appropriate form of *B. pertussis* OS. The data obtained for the oligosaccharide-comprising conjugate model could help in a rational design of neoglycoconjugate vaccines.

Presumably, the final structure of the endosomally processed carbohydrates may differ from the native antigens. It is especially important for bacterial species such as *B. pertussis*, which do not possess highly polymerized polysaccharide, but only oligosaccharides exposed on the cell surface. The processing of such oligosaccharides may undergo the same mechanism as polysaccharide molecules, however due to the lack multi-unit structure, the effect of the process may completely change the final epitope. The processing of oligosaccharides in endosomes has not been described to date. Herein, we attempt to explain some aspects of oligosaccharide vulnerability to RNS by assessing the immunogenicity of the model glycoconjugates. The structural features of *B. pertussis* 186 oligosaccharides used for the endosomal-like processing are known and thus can be correlated with the resultant specificities of anti-glycoconjugate antibodies.

## 2. Materials and Methods

### 2.1. Bacteria

*B. pertussis* strains 186 and 606 obtained from the National Institute of Public Health, Warsaw, Poland were used in this study. The bacteria were grown on a solidified charcoal-agar supplemented with sheep blood and Steiner-Scholte liquid medium for 4 days at 36 °C [35]. The bacteria were killed with 1% phenol, separated from medium by centrifugation and kept frozen for further preparations.

### 2.2. Preparation of B. pertussis Lipooligosaccharides

LOS was extracted from bacterial cells by the hot phenol-water method [36] and purify by ultracentrifugation as previously described [37]. *B. pertussis* 186 LOS was subjected to specific degradations.

### 2.3. Deamination of B. pertussis 186 LOS

The LOS was treated with nitrous acid [26,38]. Briefly, LOS (50 mg) was suspended in a freshly prepared solution (180 mL) of water/5% sodium nitrite/30% acetic acid (1:1:1, *v*/*v*/*v*) and stirred for 4 h at 24 °C. The products of LOS deamination were separated by ultracentrifugation (200,000× *g*, 2 h, 4 °C). The supernatant was freeze-dried, and the product was purified on a Bio-Gel P-2 column (1.6 × 100 cm, Bio-Rad, Hercules, CA, USA) in a 0.05M pyridine/acetic acid/water buffer, pH 5.6. The yield of the reaction was ~4 mg of pentasaccharide.

### 2.4. Isolation of B. pertussis 186 OS 

LOS (200 mg) was treated with 1.5% acetic acid (100 °C, 30 min) followed by centrifugation (40,000× *g*, 20 min). The supernatant was fractioned by a molecular sieve chromatography on TSK G3000-PW column equilibrated with water. The purified product (90 mg) was freeze-dried.

### 2.5. N-Acetylation of B. pertussis 186 OS 

OS (5 mg) was dissolved in saturated aqueous sodium bicarbonate (NaHCO_3_, 2 mL) at 0 °C. The acetic anhydride (100 µL) was added every 10 min for 30 min. The mixture was kept for another 30 min at 0 °C (on ice). The product was purified on a Bio-Gel P-4 column (Bio-Rad, 1.6 × 100 cm) and freeze-dried [39].

### 2.6. Selective Oxidation of B. pertussis 186 Oligosaccharide 

OS (20 mg) was dissolved in 0.01 M aqueous sodium periodate solution (NaIO_4_, 2 mL). The reaction was carried out in the dark at 24 °C for 1 h. The oxidation reaction was controlled by the measurement of an absorbance at 225 nm and stopped by addition of ethylene glycol (30 μL). The oxidized oligosaccharide was purified on TSK G3000-PW column and the product was freeze-dried.

All chemically pre-processed oligosaccharides, the obtained OS, N-acetylated-OS and pentasaccharide were analyzed by mass spectrometry and NMR to verify the structures.

### 2.7. Conjugation of the Oligosaccharides with the Ovalbumin Peptide (OVAp)

The peptide was obtained by *de novo* synthesis (LipoPharm, Gdansk, Poland). The oligosaccharides were coupled to OVAp by reductive amination following a previously described procedure by Jennings and Lugowski [24]. Briefly, the oligosaccharides with active aldehyde—pentasaccharide (~4 mg) and oxidized oligosaccharides (4 mg) were dissolved in 0.2 M borate buffer at pH 9.0 (250 µL, pre-warmed to 37 °C). OVAp (0.5 mg) was dissolved in 50% (*v*/*v*) DMSO in water (50 µL). The respective OS solutions were then mixed with OVAp. After one-hour incubation at 37 °C, sodium cyanoborohydride (5 M NaBCNH_3_ solution, 11 µL) and a drop of chloroform to prevent bacterial growth were added to the solution. The reaction was carried out for 14 days at 37 °C. On the 5th and 10th day after the start of the reaction, additional NaBCNH_3_ (5 M, 7.5 µL) were added. The reaction mixture was fractionated on a Bio-Gel P-4 column (Bio-Rad) equilibrated with 0.05 M pyridine/acetic acid buffer/water, pH 5.6.

### 2.8. Conjugation of the Oligosaccharides with the Pertussis Toxin (PT)

PT was isolated from *B. pertussis* 186 culture by ion exchange chromatography [40]. The conjugates were prepared using the reductive amination method by Jennings and Ługowski [24]. The oxidized oligosaccharide (10 mg) was dissolved in 1.5 mL of 0.2 M borate buffer at pH 9.0 and added to the PT solution (0.3 mL, 1 mg). The mixture was incubated for 1 h at 37 °C, then 1 M NaBCNH_3_ solution, 240 μL (ALD coupling solution, Sterogen) and a drop of chloroform were added to the solution. The reaction was carried out for 14 days at 37 °C. Additional portions of 1 M NaBCNH_3_ (160 μL) were added on the 5th and 10th days of the reaction. The mixture was fractionated on a TSK-Gel G3000-SW column (HPLC) equilibrated with phosphate-buffered saline (PBS, pH 7.5). In the collected fractions, the protein concentration was estimated by measuring absorbance at a wavelength of 280 nm. The presence of oligosaccharide was determined by dot-blot reaction using the anti-endotoxin rabbit sera obtained by immunization with a bacterial mass of *B. pertussis* 186 and with *B. pertussis* 186 LOS-derived pentasaccharide-tetanus toxoid conjugate (All polyclonal rabbit sera were from our Laboratory collection, [26,28]). The conjugates were stored with an addition of merthiolate (0.01%) at 4 °C.

### 2.9. Immunizations

Mice BALB/c were immunized on days 0, 14 and 21. The mice were injected with conjugates (5 µg) in a formulation with the selected adjuvant in PBS or the adjuvant alone in PBS (control sample). As adjuvants aluminum hydroxide (alum), *Hafnia alvei* 1200 monophosphoryl lipid A (MPL) [41], a mixture of MPL and muramyl dipeptide (MDP) were tested. The immunizations were carried out according to the procedures approved by the Local Ethical Commission for Animal Experimentation at Hirszfeld Institute of Immunology and Experimental Therapy, Polish Academy of Sciences. After completion of the vaccination, the mice blood samples were collected and levels of anti-glycoconjugate antibodies in the sera were determined.

### 2.10. Analytical Methods

ELISA and immunoblotting were used to assess the immunochemical properties of the mouse sera. ELISA, using LOS as the solid-phase antigen, was performed by a modification [24] of the method by Voller et al. [42], and immunoblotting was performed as previously described [37]. A goat anti-mouse IgG conjugated with alkaline phosphatase (Bio-Rad) was used as a second antibody. *p*-Nitrophenyl phosphate and 5-bromo-4-chloro-3-indolylphosphate (BCIP)—nitroblue tetrazolium (NBT) were used as detection systems for the ELISA and immunoblotting, respectively. SDS-PAGE analysis of LOSs was performed by method of Laemmli [43]. The gel bands were visualized by the silver staining method [44].

### 2.11. Isolation of IgG-Enriched Fraction from Sera 

Serum (550 µL) was centrifuged (3000× *g*, 15 min, 4 °C) to remove any insoluble particles. A saturated ammonium sulfate solution (450 µL) was added to the supernatant (final saturation, 45% [*v*/*v*]) and left overnight at 4 °C. The mixture was then centrifuged, the supernatant was discarded and the pellet was dissolved in PBS. The solution was dialyzed against PBS, using Microcon YM-50 filters with a 50-kDa cutoff. The protein concentration was estimated and adjusted to 12 µM by using UV absorbance at 280 nm. The IgG-enriched fractions were used for STD-NMR analysis [26].

### 2.12. Mass Spectrometry

MALDI-TOF MS of the oligosaccharides and glycoconjugates were performed with the UltrafleXtreme instrument (Bruker Daltonics, Billerica, MA, USA). Spectra were acquired in negative and positive ion modes. 2,4,6-trihydroxyacetophenone (THAP) and 2,5-dihydroxybenzoic acid (DHB) in acetonitrile with 0.1% TFA (1:1) were used as a matrix for OSs and oligosaccharides-OVAp conjugates. Preparations of oligosaccharide-PT conjugates were dialyzed against 0.1% TFA (Microcon, 10-kDa cutoff) and mixed with matrix, synapinic acid (SA) prior the MS analysis.

### 2.13. NMR Spectroscopy

NMR spectra of the oligosaccharides and neoglycopeptides were recorded on a Bruker Avance III 600 MHz spectroscope using a 5 mm inverse-detection QCI cryoprobe equipped with Z-gradient. The samples were prepared in 99.9% ^2^H_2_O solution. Spectra were acquired at 30 °C and excitation sculpting pulse sequence was applied to suppress water signals in the spectra. The data were acquired and processed using standard Bruker software. The processed spectra were assigned with the NMRFAM-SPARKY software [45]. The Saturation Transfer Difference (STD) NMR experiments were performed using pulse programs as previously described [46]. Samples (total volume, 160 μL) were prepared in 3 mm NMR tubes, using PBS made with ^2^H_2_O, pH 7.5. The antibodies (40 μM) and oligosaccharide (5 mM) were examined. The protein was irradiated at δ_H_ −0.5 ppm (on-resonance) and δ_H_ 100 ppm (off-resonance) with a train of Gaussian shaped pulses (50 ms). The saturation time was 2 s. The broad resonances of a protein were suppressed with a 20-ms spin-lock pulse. The excitation sculpting pulse sequences were used to suppress water signals in the spectra.

### 2.14. Simulation of B. pertussis 186 OS Deamination Traced by Kinetic NMR Measurement

*B. pertussis* 186 OS (0.2 mM) was added to a freshly prepared solution (80 µL) containing water, sodium nitrite (0.5%) and acetic acid (1%). The reaction was carried out in a 1.7 mm NMR tube on a Bruker Avance III 600 MHz spectroscope equipped with an inverse TXI microprobe. The course of reaction was monitored by repetitive NMR measurements—the spectra were recorded every 30 min for 3 h.

## 3. Results

### 3.1. Isolation of Oligosaccharides with the Defined Structures from B. pertussis 186 Lipooligosaccharide

LOS of *B. pertussis* is an antigen in natural infection [20]. The complete oligosaccharide of *B. pertussis* LOS contains the epitopes for the bactericidal antibodies. It includes the core oligosaccharide and a distal trisaccharide [29]. The oligosaccharides derived from such dodecasaccharide may expose different regions as immunodominant when conjugated with a protein. Such glycoconjugates would undergo processing in endosomes prior to presentation to T-cells [30,31,32,33].

**Figure 1:**
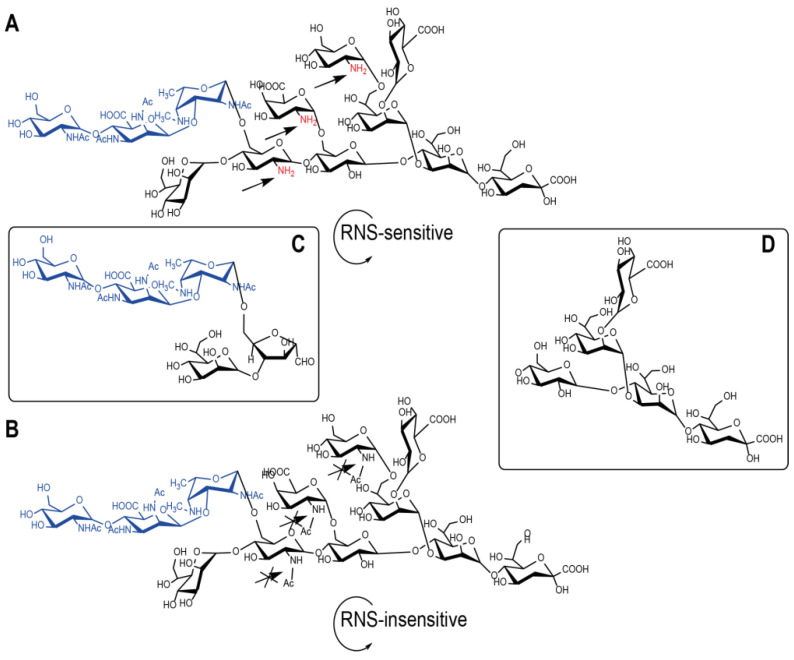
Structures of *B. pertussis* 186 LOS-derived oligosaccharides used as a model of the endosomal processing (**A**) complete native OS with free amino groups, making it vulnerable to RNS, (**B**) the N-acetylated OS form RNS-insensitive, (**C**) distal pentasaccharide, (**D**) the OS inner core segment, devoid of the pentasaccharide and the amine-bearing monosaccharides. The anticipated vulnerable structural elements and these subjected to modification by reactive nitrogen species (RNS) are indicated by colors in the structures. Arrows mark the RNS-sensitive/insensitive monosacharides.

The complete *B. pertussis* core OS contains non-acetylated glucosamine residues which are susceptible to deamination. Therefore, we have devised a model (Figure 1) that would correlate the susceptibility of *B. pertussis* 186 LOS-derived dodecasaccharide to degradation by deamination as a way to mimic the effects of the glycan processing by the nitric oxide–related RNSs in vivo.

In our project, the OS was degraded in a controlled way to obtain the oligosaccharides modified by this process in vitro. Subsequently, neoglycoconjugates of the LOS-derived oligosaccharides were prepared and their immunogenicity was assessed.

**Figure 2:**
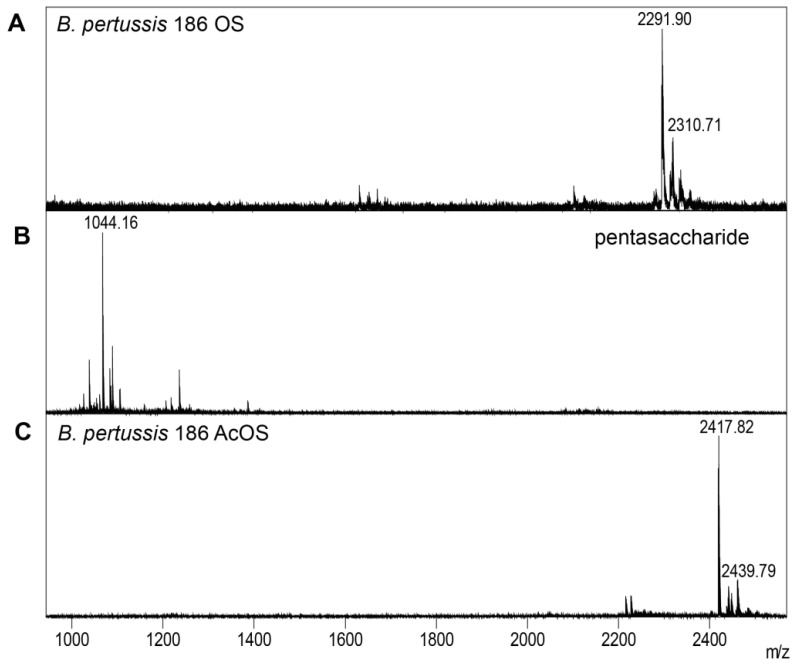
MALDI-TOF MS spectra of *B. pertussis* 186 LOS-derived oligosaccharides: (**A**) complete native OS (dodecasaccharide), (**B**) pentasaccharide isolated by the deamination of the OS and (**C**) N-acetylated OS. Spectra were recorded in negative ion mode using 2,4,6-trihydroxyacetophenone (THAP) or dihydroxybenzoic acid (DHB) in AcN/0.1% TFA (1:1) as a matrix.

Three types of LOS-derived oligosaccharides of *B. pertussis* 186 were prepared and conjugated to a peptide or protein carriers. The structures of the chemically pre-processed oligosaccharides were analyzed by mass spectrometry and NMR spectroscopy (Figure 2 and Figure S1).

Firstly, *B. pertussis* 186 LOS was hydrolyzed with 1.5% acetic acid, yielding a water-soluble oligosaccharide fraction. The product was purified and analyzed by MALDI-TOF MS. The signals at *m*/*z* 2291.90 and 2310.71 (Figure 2A) correspond to an anhydro- and dodecasaccharide, respectively (Figure 1A). Subsequently, the OS was oxidized by 0.01 M sodium periodate. The signal at *m*/*z* 2279.80 in the spectrum of the oxidized OS (data not shown) corresponded to the complete dodecasaccharide with a modification of Kdo. Under the condition of this experiment—a mild oxidation by sodium periodate only the Kdo residue is selectively oxidized. The linkage between two vicinal carbon atoms substituted by hydroxyl groups (diol) in the side chain of Kdo is cleaved and free aldehyde group is formed. The OS-oxidized constitutes the activated form of the oligosaccharide, which is suitable for conjugation to a carrier.

The second batch of *B. pertussis* 186 LOS was treated with nitrous acid, exploiting the presence of the glucosamine residues that resulted in the cleavage of the distal oligosaccharide segment. The nitrous acid caused a selective cleavage at a glycosidic bond of the →4,6)-α-d-Glc*p*N-(1→ and yielded two main oligosaccharides: inner core of the OS (Figure 1D) and a pentasaccharide (Figure 1C), comprising the distal trisaccharide. In the spectrum of the product the signal at *m*/*z* 1044.16 (Figure 2B) corresponds to the pentasaccharide. The obtained pentasaccharide mimics the potential product of endosomal processing of *B. pertussis* 186 OS by reactive nitrogen species.

The third batch of *B. pertussis* 186 LOS was used to prepare the OS form that would not be susceptible to deamination. The isolated *B. pertussis* 186 LOS-derived OS, containing glucosamine residues, was subjected to the selective N-acetylation. In the MALDI-TOF MS analysis the signals at *m*/*z* 2417.82 and 2439.79 (Figure 2C) corresponded to dodecasaccharide (complete *B. pertussis* 186 OS), with three additional N-acetyl groups (Figure 1B). This procedure makes the original OS resistant to degradation by deamination as N-acetyl groups cannot be nitrosiated by nitrosium cation [31]. The N-acetylated OS was used here to test the devised model of *B. pertussis* 186 OS endosomal processing by reactive nitrogen species. The N-acetylated OS was subsequently activated by a selective mild oxidation for conjugation by reductive amination.

The specific degradations of *B. pertussis* 186 LOS and modifications of the LOS-derived oligosaccharides produced: (1) the complete dodecasaccharide [mimicking the main most vulnerable form of the OS to endosomal RNS and ROS actions], (2) the pentasaccharide comprising the distal trisaccharide [mimicking the main product of the RNS actions] and (3) the N-acetylated OS (AcOS, Figure 1) [mimicking the main product resistant to the RNS actions].

### 3.2. Synthesis of B. pertussis 186 Oligosaccharide-Carrier Neoglycoconjugates. Structural Analysis and Immunochemical Properties of Conjugates

To mimic endosomal processing of glycoconjugates and obtain the immunological response each of the three types of LOS-derived oligosaccharides of *B. pertussis* 186 was conjugated to a carrier. Two different carriers were employed: ovalbumin peptide OVA323-339 (OVAp) and pertussis toxin (PT).

As previously described [33] the OVA323-339 is the MHC II-binding peptide which constitutes a T-cells epitope of ovalbumin. OVAp was N-acetylated at the N-terminus and extended with 4 additional amino acids as the C-terminal residue. The amino acid sequence: N-acetyl-ISQAVHAAHAEINEAGRESGK was verified by MALDI-TOF MS analysis. The C-terminal lysine was readily available for the conjugation with an aldehyde group of the activated oligosaccharides.

The oligosaccharides were conjugated to the OVA-peptide using a reductive amination method [24]. Pertussis toxin (PT) was used as an alternative carrier protein for the separate synthesis of the neoglycoconjugates with LOS-derived oligosaccharides and the same reductive amination procedure was followed. PT was prepared as described previously [28]. The products of the neoglycoconjugate synthesis were analyzed using immunochemical methods, mass spectrometry and NMR spectroscopy.

The glycoconjugates with OVAp were characterized by mass spectrometry (Figure S2). The observed ions [M + H]^+^ at *m*/*z* 4498.48 and 3246.44 corresponded to the OS-OVAp and the pentasaccharide-OVAp conjugates, respectively. The corresponding signals were not observed in spectra for the acetylated OS-OVAp conjugate, as this conjugate did not ionize efficiently. The glycoconjugates were tested with rabbit anti-*B. pertussis* OS antibodies specific for the whole cell of *B. pertussis* 186 and for the *B. pertussis* 186 pentasaccharide-tetanus toxoid conjugate as described previously [26]. The oligosaccharide-OVAp conjugates reacted with the both sera in immunoblotting (dot-blot) and ELISA.

The prepared glycoconjugates were used for immunization of mice in an attempt to model the endosomal-like pre-processing of *B. pertussis* 186 OS and to assess their immunogenicity in relation to the underlying structural features.

### 3.3. Immunizations and Analysis of Immunogenicity of B. pertussis 186 LOS-Derived OS Neoglycoconjugates

The glycoconjugates were employed to immunogenicity studies. Five schemes of immunizations (numbered I–V, Figure S3) were carried out with modifications, which were introduced to use the most optimal antigen formulation and immunization procedures. The serum samples were taken to determine the titers of anti-*B. pertussis* LOS immunoglobulin G (IgG). The sera obtained by immunization of mice with the conjugates of *B. pertussis* 186 OS, N-acetylated OS and pentasaccharide were tested with *B. pertussis* LOS of strains 186 and 606 in ELISA. The differences in immunogenicity were correlated with the hypothesized glycan-processing pathways.

In the initial immunization series (I–III) the groups of BALB/c mice were immunized subcutaneously with the glycoconjugates comprising OVAp. In the first immunization (I, 10 mice/group) animals were maintained under specific pathogen free condition (SPF) and immunized with the antigen formulated with Al(OH)_3_ adjuvant. As the initial titers of anti-*B. pertussis* 186 LOS antibodies were low, in the subsequent immunization (II) an attempt was made to select the optimal adjuvant. The alum, MPL and the MDP+MPL mixture were tested. However, no substantial booster effect was observed, and the IgG responses were similar with only marginal increase for MPL. Subsequently, MPL was used as adjuvant for further immunizations. The use of the BALB/c mice maintained under conventional conditions (non-SPF) in the immunization III did not enhance the level of antibodies and the anti-*B. pertussis* 186 LOS IgG levels in the sera raised by the studied glycoconjugates with OVAp were similarly low and no statistical difference to control sera titers was found.

Due to low immunogenicity of the oligosaccharide-OVAp conjugates, the carrier and the administration route were changed. As *B. pertussis* 186 OS-PT and pentasaccharide-PT conjugates were immunogenic in the rabbit model [28], the conjugates with pertussis toxin as a protein carrier were prepared and used in further analysis. The structures of oligosaccharides were identical to these used in the OVAp-conjugates.

In a pilot study, mice were injected intraperitoneally with antigens: penta-PT and OS-OVAp conjugates and control sample. These preliminary results demonstrated that pentasaccharide-PT conjugate induced high antibody titers against *B. pertussis* 186 LOS. The significant difference between anti-penta-PT antibodies and these of non-immune serum (*p* = 0.038) was found (immunization IV, Figure S3).

**Figure 3:**
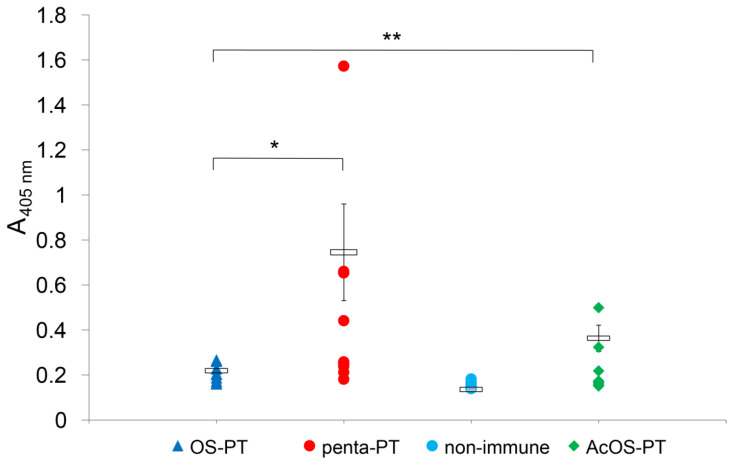
Anti-*B. pertussis* 186 LOS IgG levels in sera of mice immunized with OS-PT, penta-PT and AcOS-PT conjugates in ELISA Sera were obtained from the immunization number V (for details on all the prior immunizations see Figure S3). Non-immune sera were used as a control. Each serum was 50-fold diluted and read out using *B. pertussis* 186 LOS as a solid phase in ELISA. Each individual symbol corresponds to a single mouse serum. Horizontal bars indicate arithmetic mean of IgG titers for each group. Differences between group titers as compared to these of non-immune sera were significant at *p* < 0.05. Significant differences were also noted between the OS-PT and penta-PT (*, *p* = 0.032) as well as OS-PT and AcOS-PT (**, *p* = 0.039 Abbreviations: OS-PT—conjugate of *B. pertussis* 186 OS and pertussis toxin (PT), penta-PT—conjugate of the pentasaccharide derived from *B. pertussis* 186 with pertussis toxin, AcOS-PT –conjugate of the N-acetylated *B. pertussis* 186 OS and pertussis toxin, OVAp—ovalbumin peptide.

Afterwards, the main experiment with 10 mice in each group was performed (Figure 3 and Figure S2, immunization V). Immunization with oligosaccharide-PT conjugates revealed that the anti-*B. pertussis* 186 LOS antibody levels differed significantly in pair-wise comparisons (*p* < 0.05).

### 3.4. Immunochemical Properties of Antibodies Recognizing the Pre-Processed B. pertussis 186 Oligosaccharides

**Figure 4:**
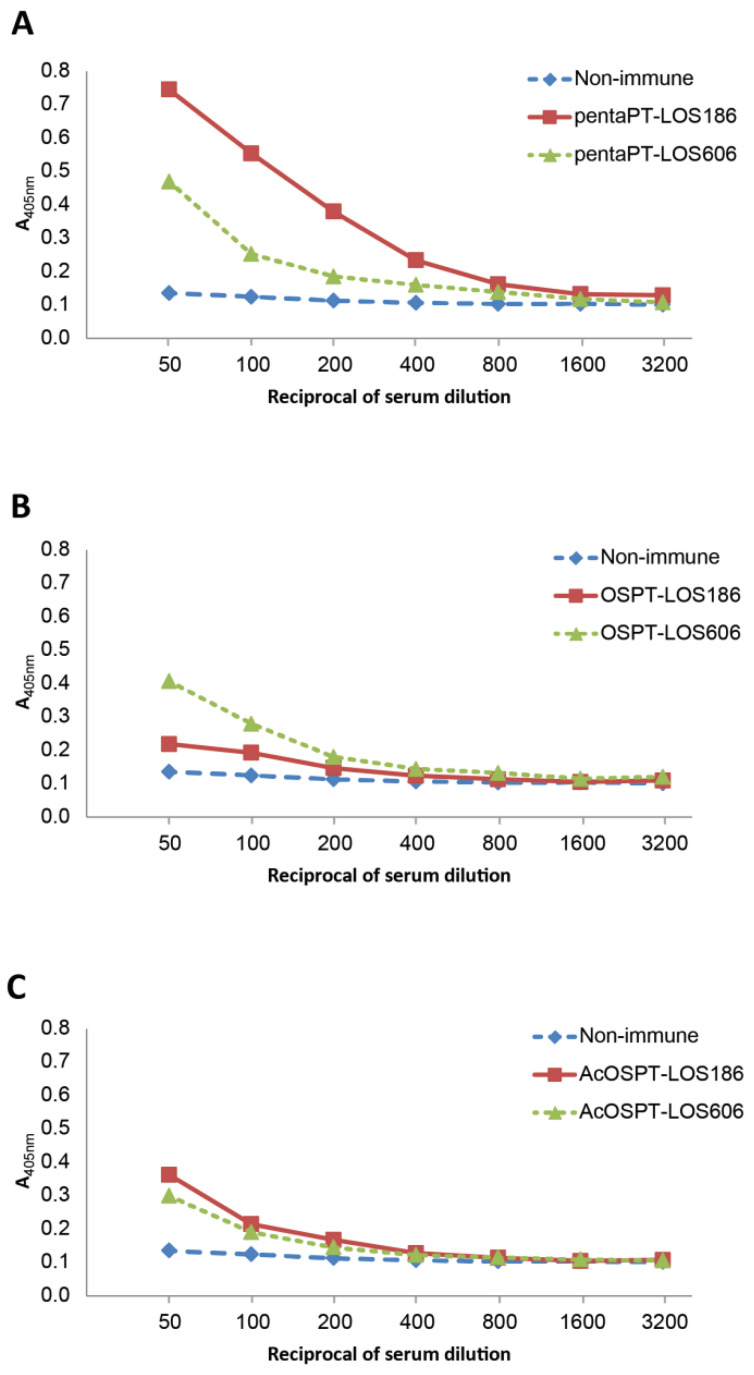
Reactivities of polyclonal antibodies against penta-PT (**A**), OS-PT (**B**), AcOS-PT (**C**) with LOS of *B. pertussis* 186 and 606 in ELISA. Non-immune sera were used as control. The mice sera (10 mice/group) were pooled for groups of antigens. A sample from a pool for each group was initially diluted 50-fold and tested as 2-fold serial dilution in ELISA. *B. pertussi*s LOS of strains 186 and 606 were used as a solid phase. LOS of strain 606 is devoid of a distal trisaccharide. The standard deviations did not exceed 10% and are not shown.

The combined results of the serological and structural analyses, and in particular comparison of immunogenicity of the glycoconjugates containing different pre-processed and complete *B. pertussis* 186 oligosaccharides were correlated (Figure 4).

The anti-*B. pertussis* 186 LOS IgG level in pooled sera raised by pentasaccharide-PT conjugate was significantly higher than this induced by OS-PT conjugate (Figure 3). The N-acetylated OS-PT induced also significantly higher IgG levels than OS-PT, but lower than the pentasaccharide-PT. To further explore the differences in specificity of the antibodies the reactivities with a nonasaccharide core OS of *B. pertussis* 606 LOS have been also investigated. Anti-OS-PT antibodies reacted strongly with *B. pertussis* 606 LOS and to a lesser extent with *B. pertussis* 186 LOS (Figure 4B). In contrast, anti-penta-PT antibodies demonstrated stronger reactivity with dodecasaccharide-comprising *B. pertussis* 186 LOS (Figure 4A). Antibodies against the N-acetylated OS-PT reacted with LOSs of *B. pertussis* 186 as well as of strain 606 to a similar extent (Figure 4C).

The interactions of anti-glycoconjugate IgGs with *B. pertussis* 186 OS were also observed in immunoblotting (Figure S3A,B), and followed by the STD-NMR (saturation transfer difference, Figure S3). STD-NMR experiments were performed to assess the specificity of anti-glycoconjugate antibodies in binding to *B. pertussis* 186 OS (Figure S4). The IgG enriched fractions from pooled mice sera were prepared for this assay. The ^1^H STD-NMR difference spectra indicated that the signals of *B. pertussis* 186 OS in the presence of the anti-pentasaccharide-PT IgGs showed higher intensity than these observed when anti-OS-PT and anti-AcOS-PT IgGs were used. The enhanced resonances belonged to N-acetyl and N-methyl groups of the distal trisaccharide, indicating that this segment of OS is involved in recognition and binding to anti-conjugate antibodies. The signal intensities in the spectra of *B. pertussis* 186 OS in the presence of anti-OS-PT IgG (Figure S4A) as well as the OS with anti-AcOS-PT IgG (Figure S4B) were comparable to these of control serum (Figure S4D). The results exhibit that only small fraction of antibodies which recognize the OS structural elements occurs in the polyclonal anti-OS-PT and anti-AcOS-PT sera. STD-NMR analysis was in agreement with data from the ELISA experiments, suggesting strongly that anti-pentasaccharide-PT antibodies are specific to the distal trisaccharide, while anti-OS-PT and anti-AcOS-PT IgGs recognize this trisaccharide only to a lesser extent (Figure 4). The pentasaccharide forms an immunodominant epitope of the *B. pertussis* LOS. It can be lost upon processing, leading to an altered immune response manifested by the modified specificity of the antibodies induced by OS-PT and AcOS-PT conjugates. Thus in agreement with the initial hypothesis the expected increased immunogenicity of the distal trisaccharide-containing pentasaccharide was confirmed.

### 3.5. In Vitro Chemical Simulation of Deamination of B. pertussis 186 LOS

**Figure 5:**
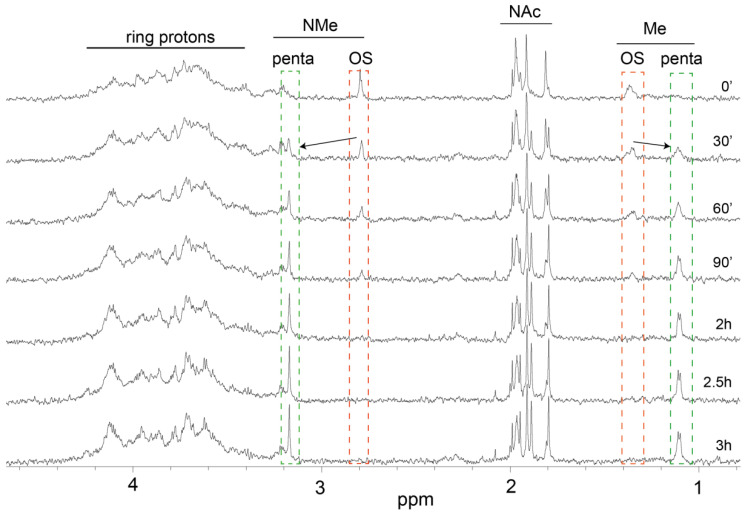
In vitro chemical simulation of *B. pertussis* 186 OS deamination traced by kinetic NMR approach. The reaction was carried out in a capillary NMR tube [∅ 1.7 mm] and the course of reaction was monitored by a series of NMR measurements with 30 min intervals for 3 h. The spectra were acquired in a pseudo-2D experiment on a Bruker Avance III 600 MHz spectroscope at 298K, using inverse TXI microprobe.

As we hypothesized that the deamination is the main endosomal processing mechanism of *B. pertussis* 186 OS we have devised a kinetic model of pre-processing of *B. pertussis* 186 OS by the treatment with NO in an in vitro chemical simulation. To mimic the mechanism of the deamination mediated by RNSs, the *B. pertussis* 186 OS was subjected to nitrous acid and the progress of the reaction was monitored in real-time by NMR (Figure 5) using the main structure reporter groups resonances to trace the structural changes in the OS.

The reaction was carried out in the NMR microtube [∅ 1.7 mm capillary tube] placed in the magnet and the reaction progress was followed by acquisition of 1D spectra every 30 min for 3 h. The signals characteristic for the native OS were disappearing while new resonances were building up in the spectra. The new resonances corresponded to these previously attributed to a distal pentasaccharide. After 30 min of the reaction the first indications of the released pentasaccharide appeared in the spectrum, and 2 h of reaction time was sufficient for the signals of the OS structure reporter groups to go below the detection limit of the experimental set-up. The positions of the observed peaks which corresponded to N-methyl (*NMe*) and deoxy (Me) groups of Fuc2*NAc*4*NMe* (indicated by arrows, Figure 5) have shifted during reaction. The observed modifications originate from the OS being degraded at the Glc*N* residues. As the result the distal trisaccharide-comprising pentasaccharide and the modified inner core oligosaccharide were formed (Figure 1C,D). After two hours of the reaction no further changes were observed, showing that the pentasaccharide constitutes the stable product of the deamination. It has also indicated that the *B. pertussis* OS is vulnerable to RNS and can yield different antigenic determinants.

## 4. Discussion

The carbohydrate epitopes are generated during endosomal processing of glycoconjugates [29,30,31,32]. The polysaccharide part can be deaminatively depolymerized by the action of free radicals such as reactive nitrogen species.

The exact pathway of oligosaccharide processing in endosomes has not been described so far. It can be assumed that the oligosaccharides may be processed by the same processes as these described for polysaccharides, but the effect on the generated epitopes can differ. As polysaccharides and complex glycans are typically composed of many repeating units, they are oxidatively depolymerized to the smaller fragments during endosomal processing [29,30,31,32]. The presence of a repetitive patterns of their structural elements provides representative determinants of the PS that remain intact and yield the carbohydrate-specific antibodies. In case of lower-sized oligosaccharides, in which the repeating units are usually not present, the loss of some labile residues due to endosomal processing may change the structures of the antigens completely and in consequence alter the specificity of generated antibodies as well.

The oligosaccharides containing the free amine groups are prone to deamination and can be degraded in endosomes. Consequently, they could be presented in the form devoid of immunodominant epitopes and then be less efficient in production of specific antibodies. In such a case, the native and complete carbohydrate structure is not an optimal antigen to induce the expected immune response. The solution is to find the immunologically active structures and design the components with desired antigenic properties.

Herein we assessed the relation between the immunogenicity and structure-preprocessing for oligosaccharide isolated from *B. pertussis* 186 lipooligosaccharide. The oligosaccharides corresponding to the hypothetical endosomally pre-processed structures were prepared and conjugated to peptide and protein carriers. Subsequently, the remote effect of the endosomal processing was measured by the immunogenicity and specificity of the anti-glycoconjugate antibodies. The *B. pertussis* 186 OS consists of the distal trisaccharide linked to inner core through amino sugar. The free amine groups are prone to deamination and therefore the terminal segment may be cleaved off from the OS. We hypothesized that the deamination constitutes the dominant processing mechanism of *B. pertussis* 186 OS. Thus the distal trisaccharide-comprising the pentasaccharide (Figure S1), the complete OS and the acetylated derivative of the OS dodecasaccharide were used to investigate the specificities of antibodies raised by immunization with their respective neoglycoconjugates (Figure 3, Figure 4 and Figure S3).

The titers of antibodies induced by the conjugates of the oligosaccharides with a peptide carrier—OVAp in the reactions with *B. pertussis* 186 LOS as a solid phase were low (Figure S2). These observations have been in contrast to the results previously described [33]. OVAp was reported as a sufficient carrier to induce strong anti-carbohydrate (polysaccharide) immune response in mice. However, our studies demonstrate that OVAp in the conjugates was suboptimal in provoking the OS-specific response in mice. Therefore, we assume that low molecular-weight oligosaccharide-peptide carrier conjugates might not trigger the immune response. Moreover, we suppose that the effect may also result from different type of carbohydrates studied, i.e., polysaccharides vs. oligosaccharides. The low immune response was amended by the application of pertussis toxin (PT) as a carrier instead. The *B. pertussis* 186 OS-PT, pentasaccharide-PT and N-acetylated OS-PT conjugates were compared. The conjugate of complete *B. pertussis* 186 OS (dodecasaccharide) with PT elicited significantly lower titers of antibodies against *B. pertussis* 186 LOS than the latter two oligosaccharides. In turn, these anti-OS-PT antibodies were able to bind to LOS of *B. pertussis* strain 606—a core oligosaccharide devoid of a distal trisaccharide. The reactivity of the anti-OS-PT IgGs to the core indicates a possible endosomal modification of the OS by removal of the distal epitope. Degradation of OS and the loss of the distal trisaccharide could be explained by deamination process induced by reactive nitrogen species (Figure 1). The glycosidic bond of the →)4,6-α-D-GlcpN-(1→ residue within the oligosaccharide is cleaved and two oligosaccharide fragments are produced: the distal trisaccharide-comprising pentasaccharide and the core oligosaccharide linked to a carrier peptide. Furthermore, only the oligosaccharide linked to a carrier peptide is presented by APC and in this form, it does not induce the high level of anti-*B. pertussis* 186 OS antibodies. Chemical feasibility of such a deamination pathway of *B. pertussis* 186 OS can be simulated in real-time observation by NMR (Figure 5) indicating that the dodecasaccharide is vulnerable to action of nitric oxide and undergoes degradation to distal pentasaccharide and inner core oligosaccharides.

The hypothesized intracellular processing of complete *B. pertussis* 186 OS was further supported by the fact that the N-acetylated OS variant induces significantly higher antibody levels in comparison to anti-OS-PT IgG titers. This derivative of the OS does not undergo deamination, as the N-acetylation artificially removes the sites prone to degradation by RNSs. Therefore, the acetylated-OS linked to a carrier is presented to APC in the complete form. However, the obtained titers were still lower than those elicited by the pentasaccharide-PT conjugate. Lower overall immunogenicity of the acetylated-OS-PT antigen may be explained by the polyclonal nature of IgGs induced for the distal trisaccharide and other regions of the core oligosaccharide. Moreover the introduced N-acetyl groups represent a new structural feature and therefore they may modify the specificity of antibody response, affecting the recognition of the native antigen.

The pentasaccharide-PT conjugate evoked the highest titers of *B. pertussis* 186 LOS-specific antibodies and thus was the most immunogenic of the analyzed glycoconjugates (Figure 3 and Figure S3). The anti-pentasaccharide-PT antibodies reacted strongly with *B. pertussis* 186 OS which indicated that the IgGs have recognized the distal trisaccharide of the OS (Figure 4A). Significantly higher titers and specificity of these antibodies indicate that the pentasaccharide was not changed in endosomes and thus the immunodominant epitope of *B. pertussis* 186 LOS—the distal trisaccharide was preserved. The observed reactivity of these antibodies with *B. pertussis* 606 LOS (Figure 4B) can be explained by the presence of the terminal heptose residues in both pentasaccharide and distal section of the *B. pertussis* 606 OS.

The specificity of anti-pentasaccharide-PT antibodies to the distal segment of *B. pertussis* 186 OS was confirmed by STD-NMR experiments as well (Figure S4). We conclude that the high titer of *B. pertussis* 186 LOS-specific antibodies raised by pentasaccharide-PT conjugate and low titer for OS-PT conjugate suggest that deamination can be the dominant endosomal processing mode of *B. pertussis* 186 OS. The oligosaccharide devoid of the distal pentasaccharide could be formed. Then, the antibodies against this immunodominant epitope are not produced what causes that the response induced by the complete OS is suboptimal. To evoke the maximal immune response the glycoconjugate of the distal trisaccharide-comprising pentasaccharide is required. As the pentasaccharide is not degraded during the intracellular processing, it elicits a strong response to *B. pertussis* 186 LOS through the production of specific antibodies against the distal trisaccharide. Therefore, it constitutes an appropriate form of LOS-derived OS component, suitable for a glycoconjugate vaccine design.

The immune response to the glycoconjugates depends on many consecutive intracellular mechanisms such as the processing by APC in endosomes, presentation to T cells and activation of B cells to the production of antibodies. We have now attempted to assess the remote effect of antigen processing—the antibodies generation. The results are consistent with published data according to which the carbohydrates in glycoproteins are modified before they constitute the epitopes for antibody induction. The research has shown that similar to polysaccharide endosomal processing mode, the deamination is one of the possible reactions the oligosaccharides undergo. The employed model indicates strongly that structural insight into the glycan segments of the would-be vaccine components is crucial not only for the design of the new glycoconjugate vaccines but also for the desired specificities of the resulting antibodies.

## 5. Conclusions

The oligosaccharides comprising the free amine groups in their structure were sensitive to deamination by reactive nitrogen species (RNS) in an in vitro chemical simulation. This observation can be easily transcribed to degradation by endosomal processing.

RNS-sensitive oligosaccharides that constitute a segment of the glycoconjugates could be presented in a modified form, devoid of immunodominant epitopes. In turn this may lead to production of antibodies with altered or unintended specificities.

The presented model indicates that structural features of the glycan segments are particularly vital for the design of new glycoconjugate vaccines. The complete carbohydrate structure becomes a prerequisite to devising the vaccine components that would contain immunologically active structures and lead to desired antigenic properties.

## Data Availability

The data presented in this study have been disclosed in the main text and the Supplementary Materials.

## Supplementary Materials

Supplementary Materials include Figures S1–S4. The following are available online at https://www.mdpi.com/article/10.3390/vaccines9060645/s1, Figure S1: The ^1^H, ^13^C HSQC spectrum and the 1D ^1^H NMR profile of the isolated pentasaccharide of *B. pertussis* 186 LOS, Figure S2: MALDI-TOF MS spectra of the OS-OVAp (A) and the pentasaccharide-OVAp (B) conjugates, Figure S3: Anti-*B. pertussis* 186 LOS IgG levels in sera of mice immunized with the conjugates of B. pertussis 186 oligosaccharides-carrier (OVAp, PT), tested in ELISA for sera dilution of 1:50, Figure S4: The STD NMR and reference ^1^H spectra of *B. pertussis* 186 dodecasaccharides in the presence of polyclonal anti-glycoconjugate antibodies obtained by immunization with OS-PT (A), AcOS-PT (B) and pentasaccharide-PT (C) conjugates. IgG-enriched fraction isolated from the non-immune serum was used as a control (D).
