# Supplementary material for: Structure and Immunogenicity of the Bordetella pertussis LOS-Derived Oligosaccharides in the Endosomal-Like Pre-Processing Mice Model
Source: Vaccines (Basel). 2021 Jun 13;9(6):645. doi: 10.3390/vaccines9060645 (PMC8231563; doi:10.3390/vaccines9060645)
Supplement: Supplementary file 1 [file vaccines-09-00645-s001.zip › vaccines-1238473-supplementary.pdf]

Supplementary Figures

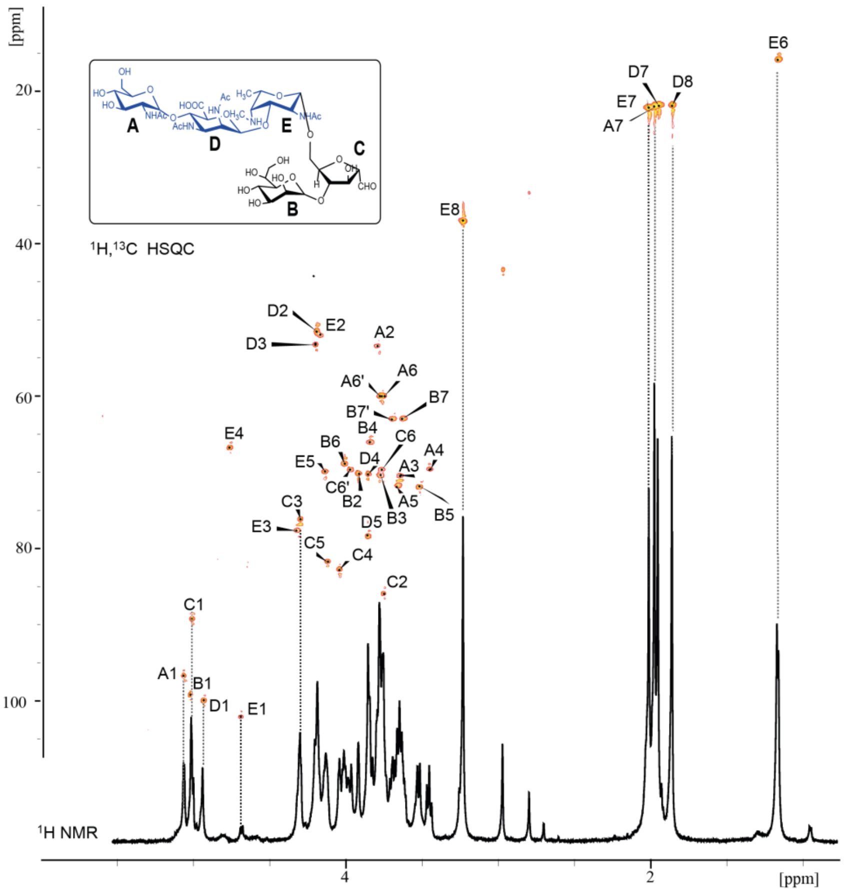

Figure S1. The ^{1}H, ^{13}C HSQC spectrum and the 1D ^{1}H NMR profile of the isolated pentasaccharide of *B. pertussis* 186 LOS. The inset shows the pentasaccharide structure. The capital letters refer to carbohydrate residues and the numbers represent proton/carbon resonances as indicated in the structure. The A7, E7, D7 and D8 signals indicate the N-Acetyl groups substituting the respective residues.

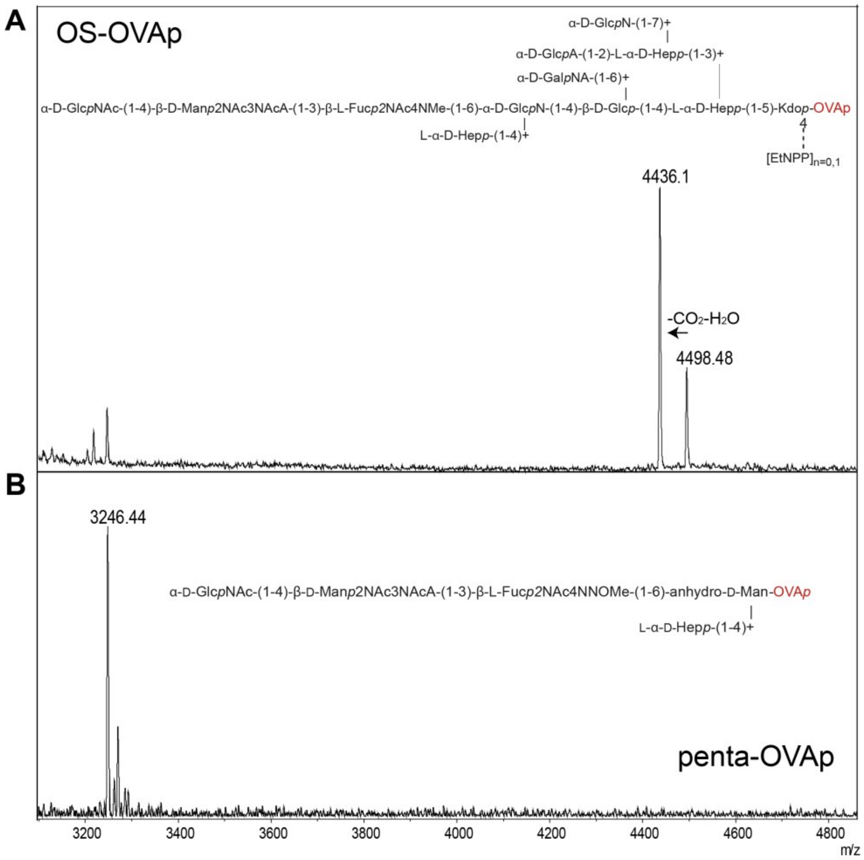

Figure S2. MALDI-TOF MS spectra of the OS-OVAp (**A**) and the pentasaccharide-OVAp (**B**) conjugates. The OS and pentasaccharides were obtained from *B. pertussis* 186 LOS. The spectra were recorded in positive ion mode using DHB as a matrix. The insets depict the structures of the neoglycoconjugates.

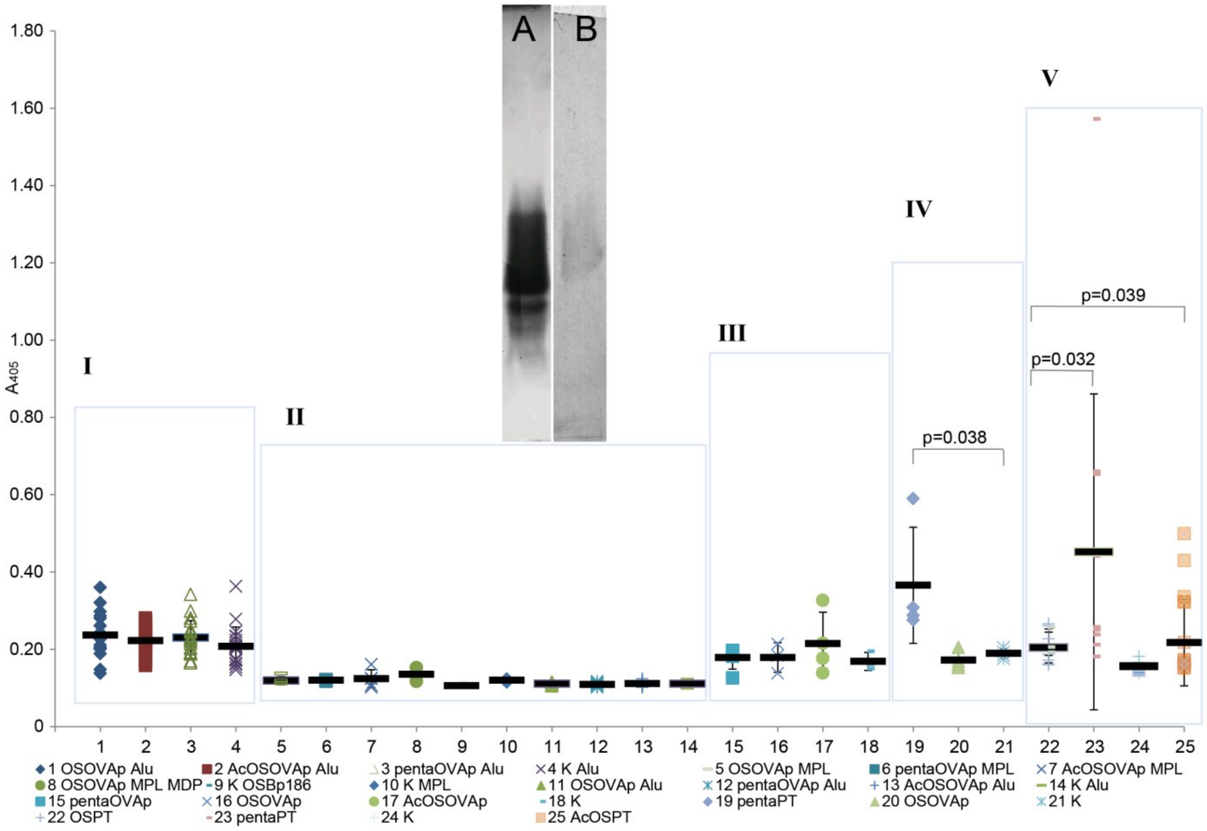

Figure S3. Anti-*B. pertussis* 186 LOS IgG levels in sera of mice immunized with the conjugates of *B. pertussis* 186 oligosaccharides-carrier (OVAp, PT), tested in ELISA for sera dilution of 1:50. I-V number represent consecutive immunizations with modifications. Each dot represents individual mouse serum. Horizontal black filled bars indicate arithmetic mean titers of each group with standard deviations showed by vertical upper and lower bars. The arithmetic mean titers which differ with 95% statistical confidence are also indicated in the chart. The inset depicts the SDS-PAGE analysis of *B. pertussis* 186 LOS (**A**) and reactivity of the anti-AcOS-PT antibodies with LOS transferred to a nitrocellulose membrane as determined by immunoblotting (**B**).

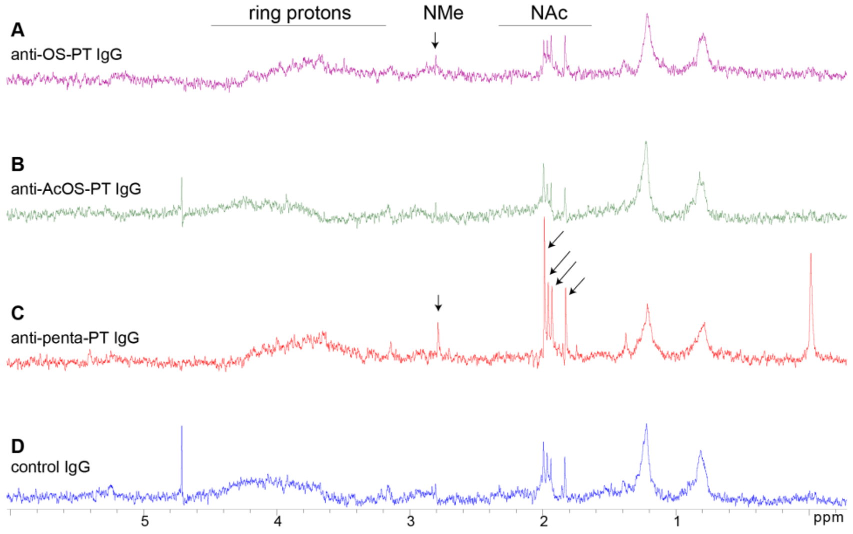

Figure S4. The STD NMR and reference ^{1}H spectra of *B. pertussis* 186 dodecasaccharides in the presence of polyclonal anti-glycoconjugate antibodies obtained by immunization with OS-PT (**A**), AcOS-PT (**B**) and pentasaccharide-PT (**C**) conjugates. IgG-enriched fraction isolated from the non-immune serum was used as a control (**D**). The Saturation Transfer Difference (STD) NMR experiments were performed using samples (total volume, 160 µl) prepared in 3 mm NMR tubes, using PBS made with ^2H_2O, pH 7.5. The antibodies (40 µM) and oligosaccharide (5 mM) were examined. The protein was irradiated at δ_H −0.5 ppm (on-resonance) and δ_H 100 ppm (off-resonance) with a train of Gaussian shaped pulses (50 ms). The saturation time was 2 s. The broad resonances of a protein were suppressed with a 20-ms spin-lock pulse. The excitation sculpting pulse sequences were used to suppress water signals in the spectra. NMR spectra were acquired at 25°C on a Bruker Avance III 600 MHz spectroscope, using 5 mm inverse detection QCI cryoprobe. The arrow symbols indicate the most pronounced resonance enhancments. For the identification of the corresponding resonances refer to Figure S4.

Abbreviations used in the descriptions of the figures: GlcNAc*,* *N*-acetyl-glucosamine; FucNAc4NMe, 2-acetamido-4-*N*-methyl-2,4,6-trideoxy-galactose; Man2NAc3NAcA, 2,3-diacetamido-2,3-dideoxymannuronic acid; Hep, heptose; Glc, glucose; GalNA, galactoaminouronic acid; GlcA, glucuronic acid; GlcN, glucosamine; Kdo, 2-keto-3-deoxy-D-*manno*-octulosonic acid; OSPT, conjugate of *B. pertussis* 186 oligosaccharide and pertussis toxin, pentaPT, conjugate of the pentasaccharide and pertussis toxin; AcOSPT, conjugate of the acetylated *B. pertussis* 186 OS and pertussis toxin; alum, aluminum hydroxide adjuvant; MPL, monophosphoryl lipid A derived from *Hafnia alvei* 1200 LPS; MDP, muramyl dipeptide; Control IgG, IgG-enriched fraction of a non-immune serum.
